# Editorial: Novel Targets and the Application of Targeting Techniques in the Treatment of Cerebrovascular Disease

**DOI:** 10.3389/fphar.2019.01359

**Published:** 2019-11-13

**Authors:** Zhidong Liu, Anna Rita Bilia

**Affiliations:** ^1^Tianjin State Key Laboratory of Modern Chinese Medicine, Tianjin University of Traditional Chinese Medicine, Tianjin, China; ^2^Department of Chemistry, University of Florence, Florence, Italy

**Keywords:** Stroke, cerebrovascular disease, cerebral ischemia, targeting, Drug delivery

Cerebrovascular diseases, also known as cerebrovascular disorder, cerebrovascular accident or cerebral vascular accident (CVA), refer to artery disease and secondary neurological deficits that is caused by dysfunction of the blood vessels supplying the brain, such as the damage and malformation of arteries. Cerebrovascular diseases can be subdivided into ischemic cerebrovascular diseases and hemorrhagic cerebrovascular disease. The most common ischemic events are vascular stenosis caused by atherosclerosis and cerebral ischemia caused by vascular occlusion. Clinical symptoms such as cerebral thrombosis and cerebral embolism are all ischemic cerebrovascular diseases. Hemorrhagic cerebrovascular diseases are mainly caused by hypertension, cerebral arteriosclerosis, tumor, etc. (Mensah et al., 2015; Matsumaru et al., 2017). The treatments against cerebrovascular diseases have made certain progress. However, surgical treatment is often accompanied by trauma and high risk of sequela, while most drugs for clinical use lack tissue specificity, which may seriously limit their extensive application. Thus, the development of novel therapies against cerebrovascular diseases is still eagerly required. In recent years, researchers have discovered some specific pathophysiological changes caused by cerebrovascular diseases, and more drug targets for cerebrovascular diseases have been successfully identified (Zhai and Luan, 2016), which makes it possible to achieve efficient therapy for cerebrovascular diseases.

This Research Topic covers some recent investigations in research on novel targets for cerebrovascular disease and the application of targeting technology for specific delivery of drugs to lesion sites, covers original research and reviews at the physiological, pharmacological and pharmaceutical levels. It contains 8 articles, including a review, from 75 authors.

Shao et al. reported a review on intracerebral hemorrhage (ICH). The authors described the mechanisms of injury after ICH and discussed some key pathophysiology mechanisms in ICH oxidative stress, inflammation, cytotoxicity of erythrocyte lysates, and neurotoxicity of thrombin. These complicated pathophysiology mechanisms are interrelated, but have not yet been fully elucidated. This review also summarized the corresponding therapeutic targets and strategies. The authors prospected a multi-target neuroprotective therapy which will make clinically effective treatment strategies possible.

Duan et al. investigated the mechanisms of action and therapeutic targets of Tao-Hong-Si-Wu decoction against neurological deficits in experimental Middle Cerebral Artery Occlusion (MCAO) through the approach of mRNA transcriptome. This Traditional Chinese Medicine (TCM) formula protected against cerebral ischemia through PI3K/AKT and Nrf2 signaling pathways, containing *Semen prunus, Flos carthami, Rehmanniae Radix Praeparata, Radix Angelicae sinensis,* and *Paeonia lactiflora*. The high throughput mRNA sequencing was used to study various therapeutic targets of the formula, in particular the induced down-regulated genes by a comparison with the data obtained after MCAO. The study provided a theoretical basis on the use of the TCM formula for prevention of neurological deficits resulting from intracerebral hemorrhage.

A further TCM formula was investigated by Liu et al. to evaluate the effects on neurogenesis against focal cerebral ischemia induced neurological deficiency. The studies TCM formula contains *Panax ginseng* root and rhizome, *Angelica sinensis* root and rhizome and *Cinnamomum cassia* stem bark. It was found that the treatment with TCM formula improved regional blood flow and infarction volume, enhanced the expression of neurogenesis mediators, and ameliorates sensorimotor functions and recognition memory.

In the study by Zheng et al., the authors investigated the effect of a further formula, Buyang Huanwu Decoction, on angiogenesis in rats after cerebral ischemia/reperfusion injury. The formulation contains *Radix Astragali, Radix Angelicae Sinensis, Radix Paeoniae Rubra, Lumbricus, Semen Persicae, Flos Carthami* and *Rhizoma Ligustici*. The formula can significantly improve neurological function score, increase the microvascular density in the boundary ischemic area and up-regulate the SIRT1/VEGF pathway against cerebral ischemic injury in rats.

Two studies report on the protection effect of two natural products after oxygen-glucose deprivation/reoxygenation (OGD/R) mimicking damage that occurs following a stroke.

The study by Zhang et al. evaluated the effects of storax, a natural resin extracted from injuring *Liquidambar orientalis* Mill, on OGD/R induced astrocytes injury and potential mechanisms. The study demonstrated that storax alleviated expression of inflammatory cytokines and protected primary cortical astrocytes injured by OGD/R, which was partially mediated by NF-κB signaling pathway activation.

In a further study by Zhao et al., cryptotanshinone, an active constituent of the root of *Salvia miltiorrhiza*, was investigated for the neuroprotective effects on cerebral ischemia/reperfusion injury using OGD/R injured neurovascular unit model *in vitro.* The results indicated that the protective mechanism of cryptotanshinone against OGD/R damage might exert *via* inhibiting neuron apoptosis and attenuating blood–brain barrier (BBB) disruption. The cryptotanshinone inhibited neuronal apoptosis possibly by blocking the activation of MAPK signaling pathways, and cryptotanshinone alleviating BBB disruption may associate with the regulation of tight junction proteins and matrix metalloproteinase (MMP)-9 expressions.

The study of Severino et al. developed a sensitive and selective LC-MS/MS method to quantify the pharmacokinetics parameters of neurounina-1, a stroke neuroprotectant, in beagle dog plasma after intravenous administration. The new analytical method was successfully applied to rapidly determine the pharmacokinetics parameters of neurounina-1 at nanomolar ranges.

In the last paper, Bilia et al. encapsulated andrographolide (AG) in human albumin nanoparticles (AG NPs) and evaluated the crossing properties of the BBB, brain distribution, and effects in TgCRND8 mice, an Alzheimer's disease mouse model. The data of immunofluorescent analyses, the step-down inhibitory avoidance test and the object recognition test indicated that AG NPs were safe and efficient vectors for brain delivery systems, and provided strong evidence for the efficacy on cognitive functions as well. The immunohistochemical analysis of GFAP-positive astrocytes in the hippocampus of Tg mice evidenced the anti-inflammatory activity of NPs after ip administration.

## Author Contributions

All authors contributed equally.

## Funding

This work was supported by the National Natural Science Foundation of China (Grant No. 81673605).

## Conflict of Interest

The authors declare that the research was conducted in the absence of any commercial or financial relationships that could be construed as a potential conflict of interest.
